# Long COVID and risk of incident cardiovascular disease: a prospective cohort study using the Multimorbidity Integrated Registry Across Care Levels in Stockholm (MIRACLE-S) cohort

**DOI:** 10.1016/j.eclinm.2026.103846

**Published:** 2026-04-01

**Authors:** Pia Lindberg, Samuel Wiqvist, Maria Juszczyk, Seika Lee, Marta A. Kisiel, Caroline Wachtler, Marcus Ståhlberg, Åsa M. Wheelock, Artur Fedorowski, Axel C. Carlsson

**Affiliations:** a Division of Immunology and Respiratory Medicine, Department of Medicine Solna, Karolinska Institutet, Stockholm, Sweden; b Department of Respiratory Medicine and Allergy and Center for Molecular Medicine, Karolinska University Hospital Solna, Solna, Sweden; c Department of Cardiology, Karolinska University Hospital, Stockholm, Sweden; d Department of Medical Sciences, Occupational and Environmental Medicine, Uppsala University, Uppsala, Sweden; e Division for Family Medicine, Department of Neurobiology, Care Sciences and Society, Karolinska Institutet, Stockholm, Sweden; f Academic Primary Health Care Centre, Region Stockholm, Sweden; g Department of Learning, Informatics, Management and Ethics, Karolinska Institutet, Stockholm, Sweden; h Department of Medicine, Karolinska Institutet, Solna, Stockholm, Sweden

**Keywords:** Long COVID cohort study, Signs and symptoms, Cardiovascular disorders, Somatic symptoms, Symptom cluster, Primary healthcare, Diagnosis

## Abstract

**Background:**

Long COVID has emerged as a global health challenge, with increasing evidence of cardiovascular sequelae. Most previous studies have focused on hospitalised cohorts, whereas cardiovascular risk in community-managed long COVID cases remains less explored. We aimed to investigate the incidence of major cardiovascular events in individuals with long COVID compared to those without long COVID in a large population-based setting.

**Methods:**

Multimorbidity Integrated Registry Across Care Levels in Stockholm (MIRACLE-S) is a population-based cohort that covers all providers of healthcare for around 2.5 million residents in Stockholm County. Individuals aged 18–65 years with a physician-assigned long COVID diagnosis (ICD-10: U09.9) between October 2020 and January 2025 were identified. Exclusion criteria were hospitalisation for acute COVID-19 or pre-existing cardiovascular disease. Cox proportional hazards models estimated the effect of long COVID on a composite cardiovascular outcome (myocardial infarction, heart failure, cardiac arrhythmias, stroke, peripheral arterial disease), adjusting for demographic, lifestyle, and mental health factors.

**Findings:**

Among 1,217,693 individuals, 8999 (0.7%) had long COVID diagnosis (66% women). Cumulative incidence of any cardiovascular event was higher in long COVID group (women 18.2%, men 20.6%) compared with control group (women 8.4%, men 11.1%). In a fully adjusted model, long COVID was associated with the composite cardiovascular outcome (women HR 2.06, 95% CI 1.92–2.22; men HR 1.33, 1.20–1.48), cardiac arrhythmia (women HR 3.11, 2.85–3.39; men HR 1.61, 1.41–1.85), and coronary artery disease (women HR 1.25, 1.04–1.52; men HR 1.26, 1.05–1.51). Heart failure incidence was elevated in women only (HR 1.25, 1.00–1.55), as also was peripheral artery disease (HR 1.25, 1.05–1.50). Long COVID was not associated with stroke in either sex.

**Interpretation:**

Long COVID is associated with increased risk of incident cardiovascular disease, particularly cardiac arrhythmias, heart failure, and coronary artery disease. These findings underscore the need for systematic follow-up and integration of long COVID into cardiovascular risk assessment.

**Funding:**

Region Stockholm and Heart Lung Foundation.

**Research in context**

**Evidence before this study**

We searched PubMed and Embase for studies published up to January 2025 using combinations of the terms “long COVID”, “post-acute sequelae of COVID-19”, “cardiovascular disease”, “arrhythmia”, “myocardial infarction”, “heart failure”, and “stroke”. Previous large cohort studies have demonstrated an increased risk of cardiovascular outcomes following COVID-19, particularly among individuals who required hospitalisation or intensive care during the acute phase. However, many studies have either combined hospitalised and non-hospitalised populations or focused predominantly on severe acute infection. Evidence on long-term cardiovascular risk specifically among non-hospitalised individuals with clinically diagnosed long COVID remains limited, and sex-specific risk patterns have been insufficiently explored in population-based settings.

**Added value of this study**

Using a large population-based register covering all levels of healthcare in Stockholm County, we examined incident cardiovascular disease among non-hospitalised adults with a physician-assigned diagnosis of long COVID compared with individuals without long COVID. By restricting the cohort to individuals without prior cardiovascular disease and analysing outcomes separately for women and men, this study provides novel evidence that long COVID is associated with an increased risk of cardiovascular disease even after mild-to-moderate acute infection. The excess risk was most pronounced for cardiac arrhythmias and coronary artery disease in both sexes, with additional associations for heart failure and peripheral arterial disease observed primarily among women. The long follow-up period and use of physician-assigned ICD-10 diagnoses strengthen the clinical relevance of the findings.

**Implications of all the available evidence**

Taken together with existing literature, our findings indicate that long COVID should be considered a clinically relevant cardiovascular risk factor not only among previously hospitalised patients but also in broader community-managed populations. The observed sex-specific patterns underscore the importance of incorporating sex-aware perspectives into long-COVID cardiovascular risk assessment and follow-up. These results support the need for heightened clinical vigilance and may inform the development of future risk stratification and follow-up strategies for individuals with long COVID in primary and specialist care.

## Introduction

Long COVID is increasingly recognised as a global public health crisis affecting an estimated 10–30% of individuals who have had an acute infection, regardless of initial disease severity, sometimes with persistent symptoms years after the initial infection.^1^^,^^2^ Although early attention focused on persistent fatigue, autonomic and respiratory symptoms, growing evidence suggests that COVID-19 may have long-term effects on the cardiovascular system, including increased risk for myocardial infarction, stroke, arrhythmias, and heart failure.^3^, ^4^, ^5^

Delayed cardiovascular complications are a concern, especially among individuals who had acute disease without the need for hospitalisation (i.e., not suffering from complications from intensive care). Large-scale cohort studies have reported elevated rates of major cardiovascular events following COVID-19, with risks persisting up to a year post-infection.^4^^,^^5^ Mechanisms proposed to explain the increased cardiovascular risk after COVID-19 include endothelial dysfunction, chronic inflammation, thrombogenicity, and myocardial injury.^6^^,^^7^ Most studies do not differentiate between hospitalised and non-hospitalised individuals, so the possible differences in their health trajectories are less explored. Because non-hospitalised and hospitalised individuals do not share the same cardiovascular risk factor profile, it is important to examine these groups separately. In this study, long COVID was operationalised as a physician-assigned diagnosis of post COVID-19 condition (ICD-10 U09.9) recorded in routine clinical care, acknowledging that this represents a pragmatic rather than a uniform clinical definition. Because hospitalised and non-hospitalised individuals with COVID-19 differ substantially in baseline cardiovascular risk, disease severity, and post-acute care trajectories, these groups should be examined separately when evaluating long-term cardiovascular outcomes. Hospitalisation during acute infection has also been identified as an independent risk factor for long COVID, particularly among men,^8^ further underscoring the importance of analysing these populations separately.

Using a large population-based prospective cohort with register data covering all levels of care in the entire Stockholm County, this study aims to investigate whether non-hospitalised individuals with a long COVID diagnosis are at increased risk of developing cardiovascular disease, defined as myocardial infarction, heart failure, cardiac arrhythmias, stroke, and peripheral arterial disease. Because both the exposure (long COVID diagnosis) and cardiovascular outcomes differ by sex, all analyses were conducted and reported separately for women and men.

## Methods

### Study design and population

The MIRACLE-S (Multimorbidity Integrated Registry Across Care Levels in Stockholm) is a cohort of adults containing data from the Stockholm County Healthcare Region, which includes a population of approximately 2.5 million people, encompassing both urban and rural areas. The region provides publicly funded comprehensive medical and health services. The Stockholm Region registers data in the purpose of reporting to national health registers, and its data is widely used for benchmarking and research, ensuring high reliability and accuracy. Since 1997, diagnoses have been categorised using the International Classification of Diseases, 10th edition (ICD-10), as defined by WHO.^9^ Healthcare data, including diagnoses, medical visits, and hospitalisation at all levels of care, are systematically recorded in the Stockholm Regional Health Care Data Warehouse known as VAL^10^; and a comprehensive dataset is available at Karolinska Institutet for research purposes under the acronym MIRACLE-S. The database has been used extensively for epidemiological research and provides reliable information on physician-assigned diagnoses, healthcare contacts, and demographics.

The present study included individuals from MIRACLE-S aged 18–65 years and residing in the Stockholm County, Sweden, during the period from October 10, 2020, to January 31, 2025. Individuals who had been hospitalised due to acute COVID-19 infection were excluded, allowing the study to focus on those with presumed mild or moderate infection not requiring hospital care or hospitalisation. Hospitalised individuals were excluded to maintain a more homogeneous study population and to avoid conflation of long-term cardiovascular risk related to long COVID with effects driven by severe acute illness or hospitalisation-related exposures. Given that hospitalisation itself is a strong predictor of long COVID and subsequent health outcomes,^8^ inclusion of both groups would complicate interpretation of long-term cardiovascular risk attributable to long COVID in community-managed individuals. A laboratory-confirmed SARS-CoV-2 infection was not required for inclusion, as population-wide testing was not available during the early phases of the pandemic in Sweden and testing data are not comprehensively captured in the MIRACLE-S/VAL registers. During the first pandemic wave in spring 2020, testing was largely restricted to healthcare workers, further limiting the availability of laboratory-confirmed infection data. Long COVID was therefore operationalised based on physician-assigned ICD-10 code U09.9 recorded in routine clinical care.^11^^,^^12^ Cohort participants with documented diagnoses of any predefined cardiovascular endpoint during the 5 years preceding study entry were also excluded to minimise confounding from pre-existing conditions. The 5-year lookback period refers to the 5 years preceding each individual's T0 (i.e., the individual-specific start of follow-up), not a fixed calendar period before October 2020. This window was chosen to capture clinically relevant pre-existing cardiovascular disease while avoiding misclassification from remote or potentially resolved conditions (Supplementary Table S4).^10^ Individuals with no continuous residency in the Stockholm Region throughout the study period or had protected identity status were excluded. Relocation and death during follow-up were treated as censoring events (Supplementary Table S5). Participants with incomplete data on key covariates were removed from the final study cohort.

### Ethics

This study was conducted in accordance with the Declaration of Helsinki and was based on pseudonymised registry data. The use of data was approved by the Swedish Ethical Review Authority (reference number 2021-01016, with amendments 2021-05735-02, 2022-06729, 2023-07166-02, and 2024-05462-02). Informed consent was waived because the study used de-identified routinely collected healthcare data.

### Procedures

Cardiovascular diagnoses were identified using ICD-10 codes (Supplementary Table S1).^9^ The diagnosis code for long COVID (post COVID-19 condition) is U09.9. We restricted the analysis to cardiovascular outcomes, including myocardial infarction, heart failure, cardiac arrhythmias, stroke, and peripheral arterial disease. Only physician-assigned ICD-10 diagnoses recorded in Stockholm Regional Health Care Data Warehouse (the VAL register) were included, ensuring that outcomes reflected clinically established cardiovascular disease rather than symptom-based or self-reported events.

Potential confounders were identified through ICD-10 codes registered in the VAL database during the 5 years preceding study entry. These included diagnoses reflecting established cardiovascular risk factors and lifestyle-related conditions: hypertension (I10–I15), diabetes mellitus (E10–E14), hyperlipidaemia (E78), obesity (E66), alcohol-related disorders (F10, K70), and nicotine dependence (F17). Additionally, common mental disorders such as depression (F32–F33) and anxiety (F40–F41) were included because they are associated not only with long COVID and cardiovascular outcomes, but also with increased healthcare use, which may influence the likelihood of diagnosing cardiovascular disease (Table 1).^13^^,^^14^ All covariates were defined as binary variables (yes/no) based on physician-assigned ICD-10 codes.

**Table 1:** Baseline characteristics by long-COVID status and sex.

	Not long COVID		Long COVID		Overall	
	W	M	W	M	W	M
	(N = 650,957)	(N = 557,737)	(N = 5907)	(N = 3092)	(N = 656,864)	(N = 645,933)
Age at entry						
Mean (SD)	40.8 (12.9)	41.7 (13.1)	45.7 (10.8)	46.6 (11.4)	40.9 (12.9)	41.8 (13.1)
Tobacco use F17						
No	649,198 (99.7)	556,040 (99.7%)	5883 (99.6%)	3081 (99.6%)	655,081 (99.7%)	559,121 (99.7%)
Yes	1759 (0.3%)	1697 (0.3%)	24 (0.4%)	11 (0.4%)	1783 (0.3%)	1708 (0.3%)
Alcohol use F10						
No	638,598 (98.1%)	540,091 (96.8%)	5804 (98.3%)	3004 (97.2%)	644,402 (98.1%)	543,095 (96.8%)
Yes	12,359 (1.9%)	17,646 (3.2%)	103 (1.7%)	88 (2.8%)	12,462 (1.9%)	17,734 (3.2%)
Obesity E66						
No	617,755 (94.9%)	539,749 (96.8%)	5437 (92.0%)	2901 (93.8%)	623,192 (94.9%)	542,650 (96.8%)
Yes	33,202 (5.1%)	17,988 (3.2%)	470 (8.0%)	191 (6.2%)	33,672 (5.1%)	18,179 (3.2%)
Hyperlipedemi E78						
No	637,654 (98.0%)	539,654 (96.8%)	5710 (96.7%)	2910 (94.1%)	643,364 (97.9%)	542,564 (96.7%)
Yes	13,303 (2.0%)	18,083 (3.2%)	197 (3.3%)	182 (5.9%)	13,500 (2.1%)	18,265 (3.3%)
Hypertension I10						
No	590,592 (90.7%)	491,114 (88.1%)	5134 (86.9%)	2494 (80.7%)	595,726 (90.7%)	493,608 (88.0%)
Yes	60,365 (9.3%)	66,623 (11.9%)	773 (13.1%)	598 (19.3%)	61,138 (9.3%)	67,221 (12.0%)
Depression F32–33						
No	581,243 (89.3%)	518,728 (93.0%)	4780 (80.9%)	2744 (88.7%)	586,023 (89.2%)	521,472 (93.0%)
Yes	69,714 (10.7%)	39,009 (7.0%)	1127 (19.1%)	348 (11.3%)	70,841 (10.8%)	39,357 (7.0%)
Anexiety F41						
No	549,232 (84.4%)	506,114 (90.7%)	4412 (74.7%)	2589 (83.7%)	553,644 (84.3%)	508,703 (90.7%)
Yes	101,725 (15.6%)	51,623 (9.3%)	1495 (25.3%)	503 (16.3%)	103,220 (15.7%)	52,126 (9.3%)
Socioeconomic covariates						
1. Higher socioeconomic status	166,710 (25.6%)	140,254 (25.1%)	1630 (27.6%)	767 (24.8%)	168,340 (25.6%)	141,021 (25.1%)
2. Middle socioeconomic status	229,990 (35.3%)	198,120 (35.5%)	2452 (41.5%)	1207 (39.0%)	232,442 (35.4%)	199,327 (35.5%)
3. Lower socioeconomic status	167,324 (25.7%)	150,012 (26.9%)	1644 (27.8%)	987 (31.9%)	168,968 (25.7%)	150,999 (26.9%)
4. Missing	86,933 (13.4%)	69,351 (12.4%)	181 (3.1%)	131 (4.2%)	87,114 (13.3%)	69,482 (12.4%)

Individuals with long COVID entered the cohort at an older age. Tobacco and alcohol use were rare and comparable across groups. Cardiometabolic comorbidity (obesity, hyperlipidaemia, hypertension, diabetes) and mental-health diagnoses (depression, anxiety) were more common—especially among women—with broadly similar socioeconomic distributions. All variables were included as covariates in sequential Cox models.

Footnote: ICD codes for covariates; Tobacco F17, Alcohol F10, Obesity E66, Hyperlipidemia E78, Diabetes E10-14, Hypertension I10, Depression F32-33, Anxiety F41.

Neighbourhood socioeconomic status was classified according to the Mosaic tool (Experian), which stratifies residential areas in the Stockholm Region into three categories: high, medium, and low socioeconomic status (SES).^15^ This variable was linked to participants via residential addresses at baseline and used as a proxy measure of individual socioeconomic position. Cases with missing SES information were categorised as unknown and excluded in sensitivity analyses.

### Statistics

A time-to-event analysis was conducted using Cox proportional hazards models to estimate the risk of developing cardiovascular outcomes in individuals diagnosed with long COVID compared to those without long COVID.

The follow-up period for each participant began at T0. For individuals with long COVID, T0 was defined as the date of first physician-assigned long COVID diagnosis (ICD-10 code: U09.9). For individuals with long COVID, T0 was defined as the date of first physician-assigned long COVID diagnosis; for individuals without long COVID, T0 was defined as cohort entry (October 10, 2020). As soon as the diagnostic code for long COVID was registered for an individual, the time from the date of the long COVID diagnosis until the end of follow-up on January 31. 2025 was considered the exposure period. The occurrence of a cardiovascular event, relocation, death of an individual also marked the end of the follow-up. Individuals who relocated or died during the follow-up period were censored at the time of the event to ensure accurate estimation of risk.

The exposed group consisted of individuals diagnosed with long COVID (ICD-10 code: U09.9), while the unexposed group included individuals without a long COVID diagnosis and no history of hospitalisation for acute COVID-19. The unexposed group comprised individuals without a physician-assigned long COVID diagnosis (ICD-10 U09.9) and without a history of hospitalisation for acute COVID-19, regardless of whether they had a recorded acute COVID-19 diagnosis. Cumulative incidence estimates represent crude, unadjusted event proportions within each group over follow-up. Between-group comparisons and risk estimates were derived from multivariable Cox proportional hazards models adjusted as specified below.

To account for potential confounders, a series of Cox proportional hazards models was constructed, incrementally adjusting for covariates:

Model A: Age and neighbourhood SES (high, medium, low) were adjusted for, as these are demographic factors that might influence the occurrence of long COVID diagnosis as well as the risk of cardiovascular disease.

Model B: In addition to age and SES, we adjusted for lifestyle factors and established cardiovascular risk factors, including registered diagnoses of alcohol and nicotine dependence as markers of smoking and high-risk consumption of alcohol, as well as diagnoses of obesity, hyperlipidaemia, hypertension, and diabetes.

Model C: This model further adjusted for depression and anxiety, as there are differences in common mental disorders between patients with and without long COVID.^8^^,^^16^

The primary outcome was the first occurrence of a predefined cardiovascular event (i.e., myocardial infarction, heart failure, stroke, cardiac arrhythmia, or peripheral artery disease). Subgroup and sensitivity analyses were conducted by age, sex, and neighbourhood SES to evaluate whether the association between long COVID and incident cardiovascular disease varied across these strata. Interaction terms between long COVID and key covariates (e.g., sex, age, comorbidities) were explored to test for effect modification. Hazard ratios (HR) and 95% CIs were calculated for all models. Proportional hazards were assessed by viewing the Kaplan-Meyer curves.

As a sensitivity analysis, a matched cohort was constructed to evaluate the robustness of the main findings. Individuals with long COVID were matched to individuals without long COVID in a 1:10 ratio based on sex, age, and neighbourhood socioeconomic status. Cox proportional hazards models corresponding to the fully adjusted model (Model C) were then repeated within the matched cohort. In addition to the covariates included in Model C, healthcare use (number of healthcare visits during the baseline period) was included to reduce potential bias related to differential healthcare-seeking behaviour.

### Role of the funding source

The funders had no role in study design, data collection, data analysis, data interpretation, or writing of the report. The corresponding author had full access to all data in the study and had final responsibility for the decision to submit for publication.

## Results

A total of 8999 individuals (prevalence 0.7% of study population; mean age 41.6 years [SD 12.9]) received a physician-assigned diagnosis of long COVID between October 2020 and January 2025, Fig. 1 and Table 1. Most diagnoses were recorded during 2021 and 2022, and as expected fewer cases were observed in late 2020, with sharply declining numbers in subsequent years (Supplementary Tables S2 and S3). Among those diagnosed, 5907 (66%) were women and 3092 (34%) were men.

**Fig. 1:**
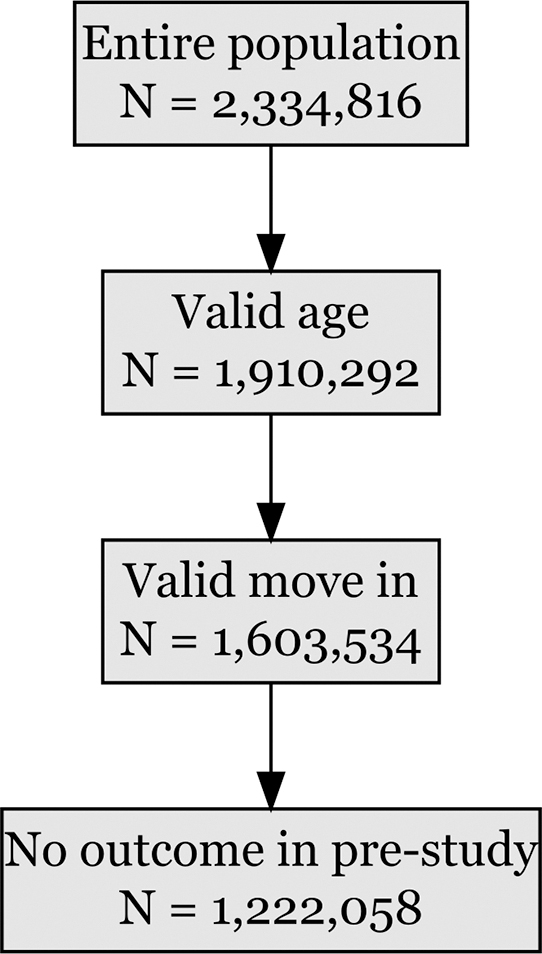
**Study population flow.** Flow diagram illustrating stepwise cohort selection from the total regional population. Numbers of individuals remaining after each exclusion step are shown, including exclusions due to age outside 18–65 years, lack of continuous residency in the Stockholm Region, and pre-existing cardiovascular outcomes during the 5-year lookback period. The final analytic cohort used for time-to-event analyses is presented at the bottom.

**Fig. 2:**
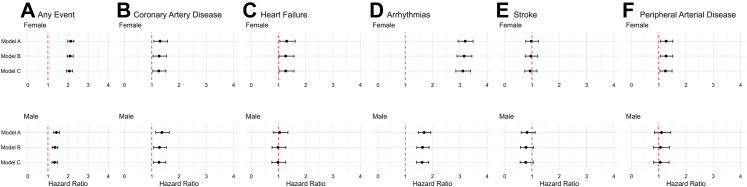
**Sex-stratified hazard ratios.** Panel 1 A shows any cardiovascular event; Panels 1 B–1 F show coronary artery disease, heart failure, arrhythmias, stroke, and peripheral arterial disease. Top row: women; bottom row: men. Estimates come from three Cox models—A adjusted for age, neighbourhood socioeconomic status; B adjusted for variables in Model A + alcohol/nicotine dependence, obesity, hyperlipidaemia, hypertension, diabetes; and C adjusted for variables in Model B + depression/anxiety. Long COVID → higher risk of arrhythmias and CAD in both sexes; heart failure and PAD in women only; stroke not significant.

**Fig. 3:**
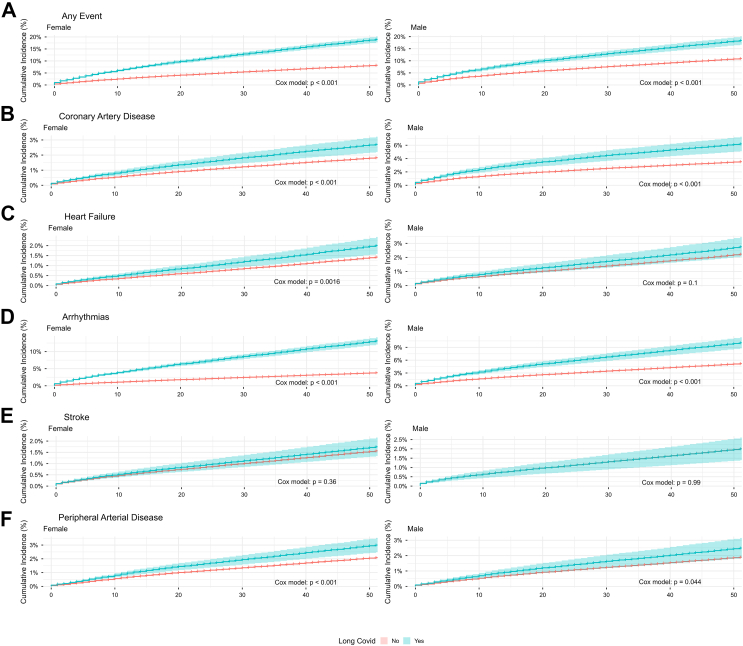
**Sex-stratified cumulative incidence by long COVID status (unadjusted).** Kaplan–Meier curves showing cumulative incidence of any cardiovascular event (2 A), coronary artery disease (2 B), heart failure (2 C), arrhythmias (2 D), stroke (2 E), and peripheral arterial disease (2 F) over ≤50 months, stratified by sex (women: left panels; men: right panels). Individuals with long COVID (blue, 95% CI shading) are compared with those without long COVID (red). Separation is most pronounced for arrhythmias and coronary artery disease in both sexes, heart failure and peripheral arterial disease primarily among women, and absent for stroke. Cox proportional hazards model p-values are shown in each panel. These cumulative incidence curves are unadjusted and based on the observed cohorts without matching and are intended for descriptive visualisation only. Risk estimates and inference are derived from the multivariable Cox regression models described in the Methods.

Among women, those with long COVID had a higher cumulative incidence of cardiovascular events compared with women without long COVID (18.2% vs 8.4%), and among men, those with long COVID also had a higher incidence compared with men without long COVID (20.6% vs 11.1%). Individuals who experienced any cardiovascular event were older (mean age 46.4 years [SD 11.6]) compared to those without events (45.9 years [SD 10.9]). In the fully adjusted model, long COVID was associated with the composite cardiovascular outcome, HR 2.06 (95% CI 1.92–2.22) in women, and HR 1.33 (95% CI 1.20–1.48) in men (Fig. 2, Fig. 3, Table 2).

**Table 2:** Cardiovascular outcomes by long-COVID status and sex.

Outcome	Not long COVID		Long COVID		Overall	
	W	M	W	M	W	M
	(N = 650,957)	(N = 557,737)	(N = 5907)	(N = 3092)	(N = 656,864)	(N = 560,829)
All cardiovascular events						
No	598,083 (91.9%)	497,315 (89.2%)	5033 (85.2%)	2664 (86.2%)	603,116 (91.8%)	499,979 (89.2%)
Yes	52,874 (8.1%)	60,422 (10.8%)	874 (14.8%)	428 (13.8%)	53,748 (8.2%)	60,850 (10.9%)
Coronary Artery Disease						
No	639,191 (98.2%)	538,221 (96.5%)	5798 (98.2%)	2959 (95.7%)	644,989 (98.2%)	541,180 (96.5%)
Yes	11,766 (1.8%)	19,516 (3.5%)	109 (1.8%)	133 (4.3%)	11,875 (1.8%)	19,649 (3.5%)
Heart_Failure						
No	641,794 (98.6%)	545,428 (97.8%)	5817 (98.5%)	3024 (97.8%)	647,611 (98.6%)	548,452 (97.8%)
Yes	9163 (1.4%)	12,309 (2.2%)	90 (1.5%)	68 (2.2%)	9253 (1.4%)	12,377 (2.2%)
Arrhythmias						
No	626,376 (96.2%)	529,080 (94.9%)	5278 (89.4%)	2854 (92.3%)	631,654 (96.2%)	531,934 (94.8%)
Yes	24,581 (3.8%)	28,657 (5.1%)	629 (10.6%)	238 (7.7%)	25,210 (3.8%)	28,895 (5.2%)
All types of Stroke						
No	640,865 (98.4%)	546,667 (98.0%)	5833 (98.7%)	3047 (98.5%)	646,698 (98.5%)	549,714 (98.0%)
Yes	10,092 (1.6%)	11,070 (2.0%)	74 (1.3%)	45 (1.5%)	10,166 (1.5%)	11,115 (2.0%)
Peripheral Arterial Disease						
No	637,483 (97.9%)	547,251 (98.1%)	5778 (97.8%)	3032 (98.1%)	643,261 (97.9%)	550,283 (98.1§%)
Yes	13,474 (2.1%)	10,486 (1.9%)	129 (2.2%)	60 (1.9%)	13,603 (2.1%)	10,546 (1.9%)

Compared with those without long COVID, the long-COVID group showed higher event rates overall, driven chiefly by arrhythmias and coronary artery disease in both sexes. Heart failure was modestly higher, most apparent in women. Peripheral arterial disease was slightly higher in women with long COVID, while stroke rates were similar between groups for both women and men. These patterns align with the sex-stratified Cox results.

Footnote: The following ICD-10 codes were used to define the outcomes: All cardiovascular events, coronary artery disease (I20–I25), heart failure I50, arrhythmias I44–49, stroke I60–69 and Peripheral Arterial Disease (PAD) I70–79.

When evaluating specific cardiovascular diagnoses, cardiac arrhythmias were markedly more common among individuals with long COVID than in those without long COVID (Fig. 2, Fig. 3, Table 2). Those diagnosed with cardiac arrhythmia had a mean age of 46.3 years (SD 13.5) compared to 41.1 years (SD 12.9) in those without. Among women, for arrhythmia, the HR for women with long COVID vs those without was 3.11 (95% CI 2.85–3.39; p < 0.0001); for men, the corresponding HR was 1.61 (1.41–1.85; p < 0.0001; Figs. 2 and 3).

For coronary artery disease (CAD), the mean age among those with CAD was 49.4 years (SD 13.0) vs 41.1 years (12.9) among those without CAD. The HR for women with vs without long COVID was 1.25 (95% CI 1.04–1.52; p = 0.021), and for men compared with men without long COVID, it was 1.26 (1.05–1.51; p = 0.014), indicating a significantly increased risk of CAD in both sexes (Figs. 2 and 3).

Individuals with heart failure were older than those without (45.9 years [SD 13.7] vs 41.3 years [13.0]). The risk estimates for heart failure among those with long COVID differed by sex. In women, the association persisted but was attenuated in adjusted models (HR 1.25, 95% CI 1.00–1.55; p = 0.047), whereas in men the association was not significant after adjustments (0.98, 0.76–1.27; p = 0.902; Figs. 2 and 3).

Peripheral arterial disease (PAD) was associated with long COVID in women but not in men. Individuals with PAD were older at entry than those without (44.9 years [SD 13.2] vs 41.2 years [13.0]). The HR for women with vs without long COVID was 1.25 (95% CI 1.05–1.50; p = 0.013), whereas among men, the association was not significant (HR 1.07, 95% CI 0.82–1.40; p = 0.618; Figs. 2 and 3). By contrast, stroke showed no statistically significant association with long COVID in either sex. Those with stroke were older than those without (47.9 years [SD 13.2] vs 41.2 years [13.0]); the HRs were 0.94 (95% CI 0.74–1.20; p = 0.630) in women and 0.77 (0.56–1.05, p = 0.093) in men (Figs. 2 and 3).

Additional analyses using propensity score-matched controls, with further adjustment for prepandemic healthcare use, yielded materially similar results. However, the association between long COVID and PAD was attenuated and no longer statistically significant (Supplementary Table S6).

## Discussion

This population-based cohort study demonstrates that individuals who developed long COVID after mild-to-moderate infection have an elevated risk of future cardiovascular disease analysed as a composite outcome. In particular, cardiac arrhythmias demonstrated markedly increased incidence in women with long COVID, although both sexes were affected. Risk of coronary artery disease was also elevated and in women and men, while heart failure and periphery artery disease were significant in woman patients with long COVID only. These findings are consistent with previous studies showing increased risk of cardiovascular sequelae in long COVID-19, including studies in non-hospitalised populations.^2^^,^^4^^,^^17^ The magnitude of excess risk for arrhythmias (HR ∼3.1 in women; HR ∼1.6 in men) is in agreement with earlier epidemiological and clinical research.^3^^,^^7^^,^^18^ These findings suggest an elevated burden of cardiovascular morbidity in individuals with long COVID, even in the absence of acute infection requiring hospitalisation.

Several mechanisms may account for the observed associations. Persistent endothelial dysfunction, autonomic dysregulation, and immune-mediated injury have all been proposed as underlying drivers of long COVID–related cardiovascular complications.^3^^,^^4^^,^^7^ The pronounced increase in arrhythmia risk, particularly among women, aligns with prior reports of elevated incidence of postural orthostatic tachycardia syndrome and cardiovascular dysautonomia in long COVID populations.^7^^,^^18^ The increased incidence of CAD and the woman-specific elevation in heart failure may reflect subclinical myocardial damage or microvascular inflammation, as suggested by imaging and mechanistic studies.^2^^,^^4^^,^^6^

The sustained elevation in risk observed across the full follow-up period, extending up to 40 months, challenges the notion of a self-limiting post-viral syndrome and supports hypotheses of long-term systemic dysfunction.^3^^,^^6^ The particularly high relative risk in younger individuals—despite lower baseline burden of cardiovascular disease—may point to greater vulnerability of intact autonomic systems or differences in care-seeking behaviour.^4^^,^^8^

It should also be acknowledged that diagnoses such as coronary artery disease and peripheral arterial disease reflect long-standing atherosclerotic processes that develop over decades.^19^ Individuals diagnosed with long COVID may therefore have had a higher burden of subclinical atherosclerosis already at baseline, which could predispose them to earlier clinical detection during follow-up. Thus, the observed associations may partly reflect differences in baseline plaque burden rather than accelerated atherosclerotic progression after long COVID. At the same time, emerging mechanistic evidence suggests that long COVID itself may contribute to vascular injury through immune-mediated pathways, including persistent complement dysregulation, thromboinflammation, endothelial dysfunction, and abnormalities of coagulation and platelet activation.^20^^,^^21^ These processes may plausibly unmask or exacerbate pre-existing asymptomatic vascular disease, thereby contributing to increased clinical detection of cardiovascular disease during follow-up. Such mechanisms are consistent with population-based evidence demonstrating elevated long-term cardiovascular risk after COVID-19, including among non-hospitalised individuals.^2^

Key strengths of this study include the use of physician-assigned ICD-10 diagnoses from a large regional health registry with complete population coverage, minimizing recall bias and enhancing diagnostic accuracy.^9^^,^^10^ The MIRACLE-S cohort provided comprehensive follow-up across care levels, allowing for robust time-to-event analyses. Additionally, our focus on individuals not requiring hospital care during the acute infection addresses an important knowledge gap, as prior studies have often concentrated on post-ICU populations with present ventilator complications.^2^^,^^4^ Unlike symptom-based surveys or patient-reported outcomes, the present study is based solely on physician-assigned ICD-10 diagnoses, allowing for objective assessment of clinically established cardiovascular conditions. However, diagnostic accuracy and consistency may vary across providers and healthcare settings, introducing potential heterogeneity in the assignment of diagnoses.

It should also be acknowledged that long COVID and cardiovascular disease may share several nonspecific symptoms, such as fatigue, dyspnoea, chest discomfort, and palpitations.^3^^,^^22^ As a result, in some individuals early or subclinical cardiovascular disease may have contributed to symptoms that led to a long COVID diagnosis, rather than the cardiovascular condition occurring strictly as a consequence of long COVID.^23^ This raises the possibility of diagnostic overlap and reverse temporality in a subset of cases, and causal interpretations should therefore be made with caution. However, the persistence of elevated risk over prolonged follow-up and after multivariable adjustment suggests that the observed associations are unlikely to be explained solely by such bias.

However, limitations should be acknowledged. Underdiagnosis of milder long COVID cases, particularly during early phases of the pandemic, may have led to conservative risk estimates. The ICD-10 code for post-COVID-19 condition (U09.9) was introduced in October 2020, and its clinical uptake likely increased gradually during late 2020 and early 2021. This may have led to under-ascertainment or misclassification of long COVID cases early in the study period. Such misclassification would be expected to dilute associations and bias results toward the null, suggesting that our estimates may be conservative with respect to this limitation. Individuals diagnosed with long COVID may also have had more frequent healthcare contacts, potentially increasing the likelihood of detecting cardiovascular disease (detection bias). Women may be more likely than men to seek healthcare for persistent symptoms and to receive a long COVID diagnosis, which could lead to differential diagnostic capture by sex. As a result, part of the observed sex difference in long COVID prevalence and associated cardiovascular risk may reflect healthcare-seeking behaviour and clinical recognition rather than biological susceptibility alone. Although our analyses were stratified by sex and thus compare individuals within the same sex, residual sex-specific diagnostic and detection bias cannot be fully excluded and should be considered when interpreting the results. The registry does not capture certain lifestyle factors (e.g., physical inactivity, diet), vaccination status, or over-the-counter medication use—unmeasured confounders that may partially influence outcomes.^6^^,^^8^ Information on the number and timing of repeated SARS-CoV-2 infections was not available in the MIRACLE-S/VAL registers, as laboratory-confirmed infection data are not comprehensively captured. Consequently, we were unable to account for multiple infections or reinfections in the analyses, which may have contributed to heterogeneity in exposure severity and long-term risk. Vaccination was rolled out in parallel with the emergence and clinical recognition of long COVID, making it difficult to disentangle its independent effects from diagnostic timing in this observational setting.^24^ Moreover, vaccination coverage in the adult population in Stockholm exceeded approximately 80% during most of the study period, suggesting limited variability and reducing the likelihood that differential vaccination alone explains the observed associations. Because this cohort is derived from routine healthcare registers, it is enriched for individuals with healthcare contact and a higher baseline burden of comorbidity than the general population. This selection may inflate absolute event rates and limits the generalisability of absolute risks to the broader community. However, relative risk estimates are less sensitive to this form of selection and remain informative for etiologic inference. Although we excluded individuals with prior cardiovascular disease, subclinical conditions may still have contributed to observed events. Nevertheless, the key associations—total cardiovascular events, arrhythmias and CAD in both sexes, heart failure and PAD in women—remained significant after multivariable adjustment.

Long COVID does not have a single, universally accepted clinical definition, and in this study the exposure was based on a physician-assigned ICD-10 code (U09.9) recorded in routine care. This may introduce heterogeneity and misclassification, as diagnostic practices may vary between clinicians, over time, and across care settings. Some individuals with persistent post-COVID symptoms may not receive a formal diagnosis, while others may be diagnosed based on differing clinical thresholds. This limitation should be considered when interpreting the findings.

Finally, the study population covers a large, diverse cohort within a publicly funded, universal healthcare system with full registry integration. This enhances the generalisability of our findings to similar high-income settings with comprehensive care access.^9^^,^^10^ However, caution is needed when extrapolating to regions with limited testing, lower baseline cardiovascular care, or different COVID-19 variant exposure. Further validation is warranted in global contexts with varying demographic and socioeconomic profiles.^17^

Although the cohort is population-based, it is derived from routine healthcare registers and therefore enriched for individuals with healthcare contact. This may contribute to higher observed event rates compared with estimates from strictly population-screened cohorts. However, in Region Stockholm approximately 90% of adults are registered with a primary healthcare provider, reflecting broad population coverage within the publicly funded healthcare system and limiting—though not eliminating—this form of selection bias.^25^^,^^26^

These findings suggest that long COVID is not a benign or transient condition, even among individuals who were never hospitalised during the primary infection. The heightened risk of future cardiovascular events, cardiac arrhythmias and CAD in both sexes, and heart failure and PAD in women—call for increased clinical vigilance. Primary care and cardiology services may consider implementing structured follow-up programs for individuals with long COVID, particularly women and younger adults, who appear to have disproportionately higher relative risk.^3^^,^^7^^,^^16^

While absolute risks remain modest at the population level, the relative hazards (≈1.3–3.1) are clinically meaningful and approach those reported for several established cardiovascular risk factors.^27^

Our results also suggest that cardiovascular risk screening of individuals with long COVID should not be limited to previously hospitalised patients but may be warranted in broader community settings. Long COVID should be integrated into cardiovascular risk assessment frameworks and health system planning,^2^^,^^17^ with particular attention to sex-specific patterns of risk.

In this population-based cohort, long COVID after mild-to-moderate infection was associated with a substantially increased risk of new-onset cardiovascular disease—most pronounced for cardiac arrhythmias and coronary artery disease in both sexes, with additional excess risk of heart failure and peripheral arterial disease in women—and these associations persisted after multivariable adjustment. Taken together, our findings position long COVID as a clinically relevant, independent cardiovascular risk factor even in younger, previously healthy adults, and they argue for structured, sex-aware follow-up and preventive strategies in post-COVID care, particularly given that cardiovascular disease in women often presents with more diffuse and atypical symptoms, which may complicate early recognition and risk assessment.

## Contributors

Study concepts: ACC, AF, PL.

Data acquisition: ACC.

Quality control of data and algorithms: ACC, AF, PL, SW.

Data analysis and interpretation: all authors.

Statistical analysis: SW, PL, ACC, AF.

Manuscript preparation: PL, ACC, AF.

Manuscript editing: all authors.

Manuscript review: all authors.

## Data sharing statement

The data used in the present study are available for research purposes after ethical approval from Stockholm Region by emailing halsodata.rst@regionstockholm.se.

## Declaration of interests

SW, SL, MJ, MAK, MS, ÅMW and AF report compensation from LINK Medical unrelated to present study.
