# Supplementary material for: Long COVID and risk of incident cardiovascular disease: a prospective cohort study using the Multimorbidity Integrated Registry Across Care Levels in Stockholm (MIRACLE-S) cohort
Source: eClinicalMedicine. 2026 Apr 1;94:103846. doi: 10.1016/j.eclinm.2026.103846 (PMC13133534; doi:10.1016/j.eclinm.2026.103846)

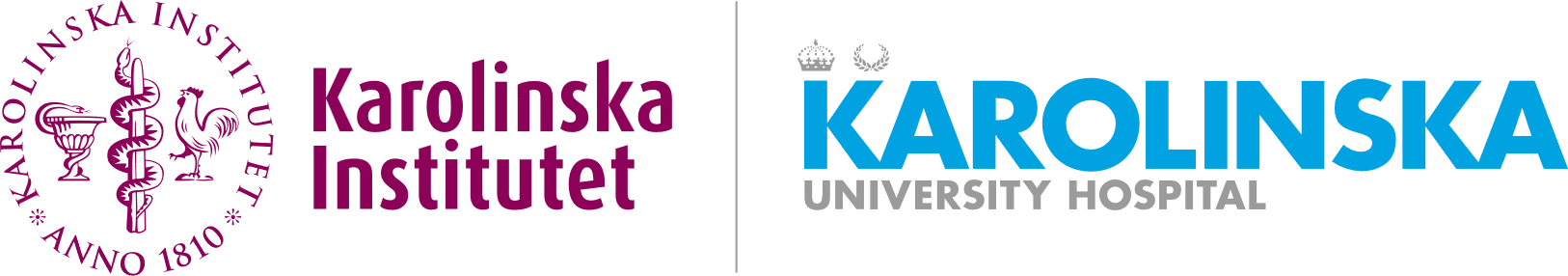


**Supplementary tables**

**Supplementary Table 1**

Outcomes and covariates were defined using physician-assigned ICD-10 codes recorded in routine clinical care. Outcomes included major cardiovascular diagnoses, and covariates comprised established cardiometabolic and mental health conditions.

| **Outcome** | **ICD Code** |
| --- | --- |
| *Coronary Artery Disease (CAD)* | I20-25 |
| *Heart Failure* | *I50* |
| *Arrhythmias* | I44-49 |
| *All types of Stroke* | I60-69 |
| *Peripheral Arterial Disease (PAD)* | I70-79 |
| **Covariates** |  |
| *Tobacco use* | F17 |
| *Alcohol use* | F10 |
| *Obesity* | E66 |
| *Hyperlipedemi* | E78 |
| *Diabetes* | E10-14 |
| *Hypertension* | I10 |
| *Depression* | F32-33 |
| *Anexiety* | F41 |

**Supplementary Table 2**

Quarterly incidence and cumulative proportion of women diagnosed with long COVID (ICD-10 U09.9) during the study period. The table shows the number of new diagnoses per calendar quarter, cumulative counts, and the corresponding cumulative incidence expressed as proportions and percentages, with the highest number of new diagnoses observed during 2021 and early 2022.

| **Quarterly incidence and cumulative proportion of women diagnosed with long COVID** | | | | | |  |
| --- | --- | --- | --- | --- | --- | --- |
|  |  |  |  |  |  |  |
| **Timeperiod** | | **n=** | **Cumulativ n=** | **Cumulative incidens** | **Cumulative incidens %** |  |
| **2020-10-01** | **Q4** | 555 | 555 | 0,093956323 | 9.4 |  |
| **2021-01-01** | **Q1** | 1380 | 1935 | 0,32757745 | 32.8 |  |
| **2021-04-01** | **Q2** | 1134 | 3069 | 0,519553073 | 52.0 |  |
| **2021-07-01** | **Q3** | 408 | 3477 | 0,588623667 | 58.9 |  |
| **2021-10-01** | **Q4** | 329 | 3806 | 0,644320298 | 64.4 |  |
| **2022-01-01** | **Q1** | 820 | 4626 | 0,783138649 | 78.3 |  |
| **2022-04-01** | **Q2** | 260 | 4886 | 0,827154224 | 82.7 |  |
| **2022-07-01** | **Q3** | 215 | 5101 | 0,863551718 | 86.4 |  |
| **2022-10-01** | **Q4** | 209 | 5310 | 0,898933469 | 89.9 |  |
| **2023-01-01** | **Q1** | 164 | 5474 | 0,926697139 | 92.7 |  |
| **2023-04-01** | **Q2** | 85 | 5559 | 0,941086846 | 94.1 |  |
| **2023-07-01** | **Q3** | 50 | 5609 | 0,94955138 | 95.0 |  |
| **2023-10-01** | **Q4** | 93 | 5702 | 0,965295412 | 96.5 |  |
| **2024-01-01** | **Q1** | 81 | 5783 | 0,979007957 | 97.9 |  |
| **2024-04-01** | **Q2** | 36 | 5819 | 0,985102421 | 98.5 |  |
| **2024-07-01** | **Q3** | 42 | 5861 | 0,992212629 | 99.2 |  |
| **2024-10-01** | **Q4** | 31 | 5892 | 0,99746064 | 99.7 |  |
| **2025-01-01** | **Q1** | 15 | 5907 | 1 | 100 |  |

**Supplementary Table 3**

Quarterly incidence and cumulative proportion of men diagnosed with long COVID (ICD-10 U09.9) during the study period. The table shows the number of new diagnoses per calendar quarter, cumulative counts, and the corresponding cumulative incidence expressed as proportions and percentages, with the highest number of new diagnoses observed in 2021 and early 2022.

| **Quarterly incidence and cumulative proportion of men diagnosed with long COVID** | | | | | |  |
| --- | --- | --- | --- | --- | --- | --- |
|  |  |  |  |  |  |  |
| **Timeperiod** | | **n=** | **Cumulativ n=** | **Cumulative incidens** | **Cumulative incidens %** |  |
| **2020-10-01** | **Q4** | 307 | 307 | 0,099288486 | 10.0 |  |
| **2021-01-01** | **Q1** | 686 | 993 | 0,321151358 | 32.1 |  |
| **2021-04-01** | **Q2** | 655 | 1648 | 0,532988357 | 53.3 |  |
| **2021-07-01** | **Q3** | 216 | 1864 | 0,602846054 | 60.2 |  |
| **2021-10-01** | **Q4** | 204 | 2068 | 0,668822768 | 66.9 |  |
| **2022-01-01** | **Q1** | 391 | 2459 | 0,795278137 | 79.5 |  |
| **2022-04-01** | **Q2** | 141 | 2600 | 0,84087969 | 84.1 |  |
| **2022-07-01** | **Q3** | 101 | 2701 | 0,873544631 | 87.4 |  |
| **2022-10-01** | **Q4** | 110 | 2811 | 0,90912031 | 90.9 |  |
| **2023-01-01** | **Q1** | 82 | 2893 | 0,935640362 | 93.6 |  |
| **2023-04-01** | **Q2** | 37 | 2930 | 0,947606727 | 94.8 |  |
| **2023-07-01** | **Q3** | 30 | 2960 | 0,957309185 | 95.7 |  |
| **2023-10-01** | **Q4** | 52 | 3012 | 0,974126779 | 97.4 |  |
| **2024-01-01** | **Q1** | 37 | 3049 | 0,986093144 | 98.6 |  |
| **2024-04-01** | **Q2** | 14 | 3063 | 0,990620957 | 99.1 |  |
| **2024-07-01** | **Q3** | 11 | 3074 | 0,994178525 | 99.4 |  |
| **2024-10-01** | **Q4** | 15 | 3089 | 0,999029754 | 99.9 |  |
| **2025-01-01** | **Q1** | 3 | 3092 | 1 | 100 |  |

**Supplementary Table 4**

Follow-up time in months (means, (SD), defined as time from T0 to cardiovascular event, death, relocation, or end of follow-up, presented by long COVID status and sex.

**Supplementary Table 5**

Distribution of censoring status during follow-up by long COVID status and sex. Values are n (%). Death and relocation were treated as censoring events; “Missing” indicates individuals without a censoring event during follow-up

**Supplementary Table 6**

Primary and sensitivity analyses of cardiovascular outcomes associated with long COVID.
Hazard ratios (estimate), p-values, and 95% confidence intervals from the primary fully adjusted model (Model C), matched analyses, and sensitivity analyses. Overall patterns were consistent across analyses, showing elevated risks of any cardiovascular event, arrhythmias, and coronary artery disease in individuals with long COVID, while associations for peripheral arterial disease were attenuated in sensitivity analyses.


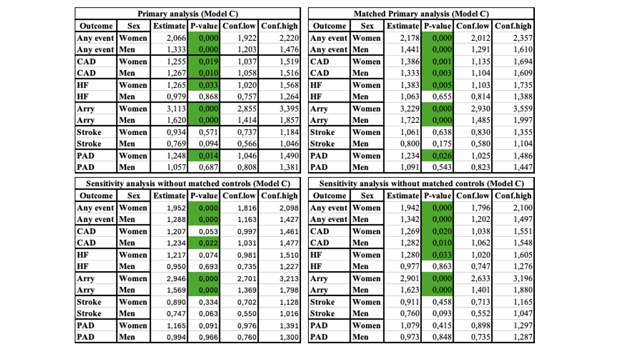

Supplement: Supplementary Tables [file mmc1.docx]
